# Comprehensive evaluation of demographic, socio-economic and other associated risk factors affecting the occurrence of dengue incidence among Colombo and Kandy Districts of Sri Lanka: a cross-sectional study

**DOI:** 10.1186/s13071-018-3060-9

**Published:** 2018-08-24

**Authors:** Lahiru Udayanga, Nayana Gunathilaka, Mohamed Cassim Mohamed Iqbal, Kosala Lakmal, Upali S. Amarasinghe, Wimaladharma Abeyewickreme

**Affiliations:** 1 Molecular Medicine Unit, Faculty of Medicine, University of Kelaniya, Ragama, Sri Lanka; 2 Department of Biosystems Engineering, Faculty of Agriculture & Plantation Management, Wayamba University of Sri Lanka, Makadura, Sri Lanka; 3 Department of Parasitology, Faculty of Medicine, University of Kelaniya, Ragama, Sri Lanka; 4 National Institute of Fundamental Studies, Kandy, Sri Lanka; 5 Regional Director of Health Services Office-Western Province, Colombo, Sri Lanka; 6 Department of Zoology and Environment Management, Faculty of Science, University of Kelaniya, Kelaniya, Sri Lanka; 7 Department of Paraclinical Science, Faculty of Medicine, Sir John Kotelawala Defense University, Ratmalana, Sri Lanka

**Keywords:** Dengue, Knowledge attitudes and practices, Socio-economic, Risk factors, Sri Lanka

## Abstract

**Background:**

Comprehensive understanding of risk factors related to socio-economic and demographic status and knowledge, attitudes and practices (KAP) of local communities play a key role in the design and implementation of community-based vector management programmes, along with the identification of gaps in existing control activities.

**Methods:**

A total of 10 Medical Officers of Health (MOH) areas recording high dengue incidence over the last five years were selected from Colombo (*n* = 5) and Kandy (*n* = 5) Districts, Sri Lanka. From each MOH area, 200 houses reporting past dengue incidence were selected randomly as test group (*n* = 1000 for each district) based on the dengue case records available at relevant MOH offices. Information on socio-economic and demographic status and knowledge, attitudes and practices were gathered using an interviewer administered questionnaire. The control group contained 200 households from each MOH area that had not reported any dengue case and the same questionnaire was used for the assessment (*n* = 1000 for each district). Statistical comparisons between the test and control groups were carried out using the Chi-square test of independence, cluster analysis, analysis of similarities (ANOSIM) and multi-dimensional scaling (MDS) analysis.

**Results:**

Significant differences among the test and control groups in terms of basic demographic and socio-economic factors, living standards, knowledge, attitude and practices, were recognized (*P* < 0.05 at 95% level of confidence). The test group indicated similar risk factors, while the control group also shared more or less similar characteristics as depicted by the findings of cluster analysis and ANOSIM. Findings of the present study highlight the importance of further improvement in community education, motivation and communication gaps, proper coordination and integration of control programmes with relevant entities. Key infrastructural risk factors such as urbanization and waste collection, should be further improved, while vector controlling entities should focus more on the actual conditions represented by the public on knowledge, attitudes and personal protective practices.

**Conclusions:**

The design of flexible and community friendly intervention programmes to ensure the efficacy and sustainability of controlling dengue vectors through community based integrated vector management strategies, is recommended.

## Background

Dengue is a vector-borne viral infection transmitted by *Aedes aegypti* and *Ae*. *albopictus* mosquitoes [1]. At present, the number of countries reporting dengue epidemics on a regular basis has increased from nine in 1970 to more than 128 countries within the past four decades, making dengue fever the most rapidly spreading mosquito-borne viral disease in the world [2]. The global burden of dengue could be ascertained by the reporting of approximately 390 million infections of dengue per year [3, 4]. The situation is more severe as a major public health issue among tropical and subtropical regions in the world.

In Sri Lanka, the most severe epidemic of dengue was recorded in the 2017 with 186,101 suspected cases [5]. Every year, a considerable amount of the annual budget is allocated in the health sector for curative and preventive measures of dengue, considering it as one of the main public health concerns in the country. Similar to many Asian countries, a complex interplay of multiple factors, e.g. urbanization, sanitation, mosquito control, meteorological, environmental, biological and demographic factors, results in dengue occurrence and transmission. Studying these factors is important in recognizing significant spatial and temporal trends of dengue outbreaks in any country [4, 6–8]. However, the relative importance of each factor on epidemic incidence and geographical distribution of epidemics may vary from one country to another, depending on the specific climatic, environmental, socio-cultural and economic conditions [9].

Regardless of the promising progress of the development and clinical evaluation of a vaccine, no vaccine or specific therapeutic treatments are yet available for dengue. This leaves the option of controlling and limiting the abundance of mosquito vector populations, as the potential solution for management of dengue epidemics [10]. After realizing the limited feasibility of chemical based vector control programmes, the Vector Controlling Entities (VCE) in Sri Lanka, are now focusing on the implementation of Integrated Vector Management (IVM) approaches. Community-based vector reduction programmes are supported as a key step under IVM as recommended by the World Health Organization (WHO) and Centers for Disease Control and Prevention (CDC) [11, 12]. However, in order to design and prior implementation of such community-based vector management programmes, it is a requisite to have an in-depth understanding on risk factors such as socio-economic and demographic factors, along with knowledge, attitudes and practices (KAP) related to dengue at local settings [13].

Studies that characterize socio-economic and demographic risk factors of populations at high and low dengue prevalence rates may enable the identification of key thematic areas to be focused in vector control, while highlighting the gaps in existing control activities [14]. However, comprehensive studies of that nature are very limited in Sri Lanka. Therefore, assessment of awareness, attitudes, practices of the local communities and associated socio-demographic risk factors of dengue, is of paramount importance for implementing community-based control programmes. Hence, the present study was designed and carried out in order to characterize socio-economic, demographic, living standards and KAP-related risk factors that affect dengue transmission in two high risk populations residing in lowland and highland areas of Sri Lanka.

## Methods

### Study design: selection of locations

**Fig. 1:**
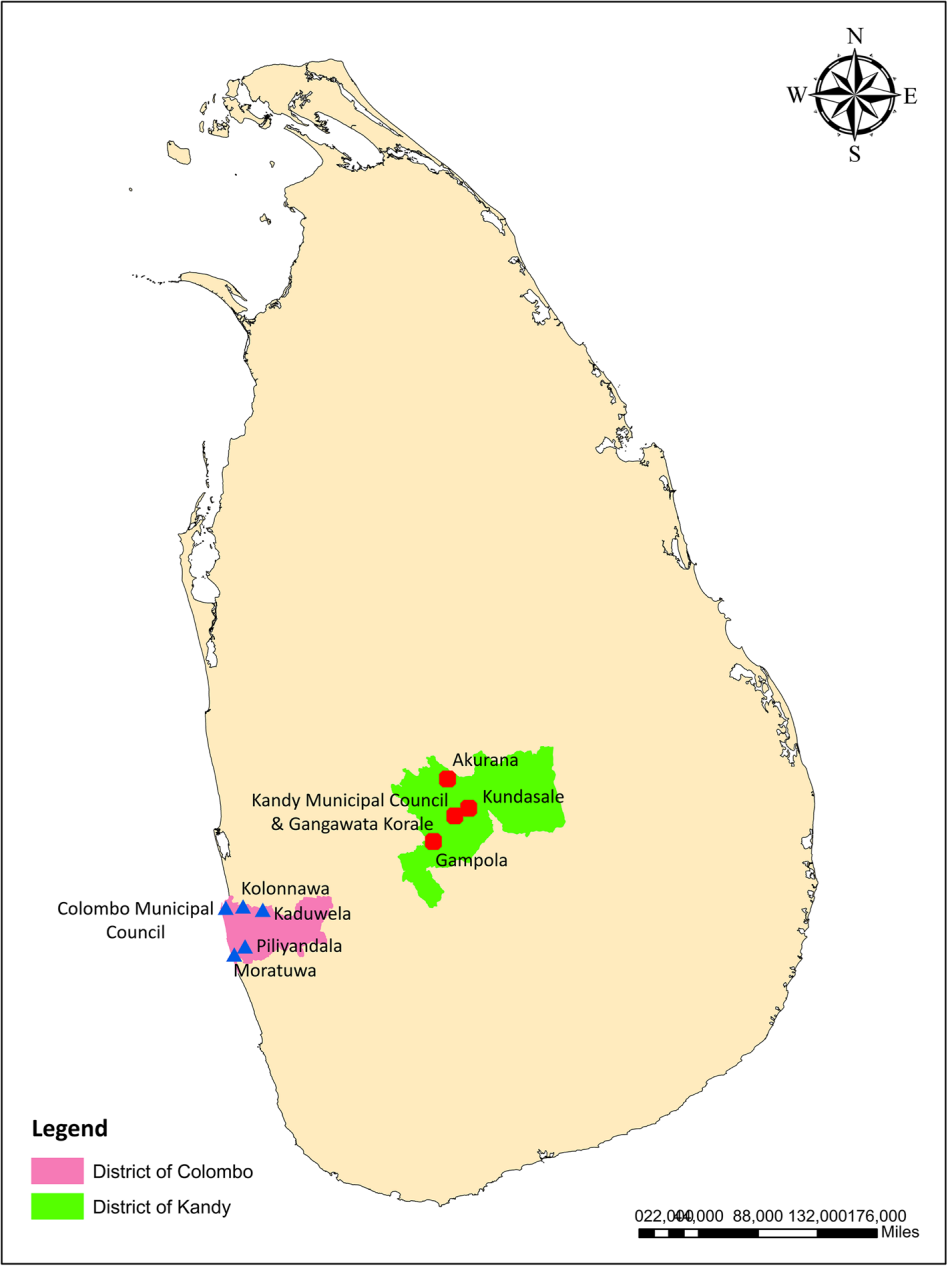
Map of the selected MOH areas in the districts of Kandy and Colombo, Sri Lanka

Colombo District (6.70° to 6.98°N and 79.83° to 80.22°E), the commercial capital of Sri Lanka, accounted for approximately 18.42% (*n* = 34,274) of the dengue cases reported from Sri Lanka in 2017 and is the most high-risk area for dengue incidence in Sri Lanka [5]. Being located in the lowland of the country, Colombo is the most urbanized metropolitan area of the country, which hosts a highly variable multi-culture and multi-ethnic population of 2,309,809 within an area of 699 km^2^ [15]. In contrast, Kandy District (6.93° to 7.50°N and 80.43° to 81.04°E), located in the central highlands (Fig. 1), extends over an area of 1940 km^2^, covering a wide array of natural environmental features in contrast to Colombo. It is of major tourist interest due to its natural location, and places of historical and religious importance. At present, Kandy District is the third highest risk area for dengue transmission in the country, contributing to 7.73 % (*n* = 14,378) of the total dengue cases reported in 2017 in Sri Lanka [5].

In this context, the study focused on socio-economic risk characterization of highland and lowland populations of the country with notable variations in land use, demography, living standards and KAPs. The lowland areas included in this study were predominantly urban areas compared to the semi-urban and rural highland areas (except for the Kandy Municipal Council area) in the highland. From each district, five high-risk Medical Officer of Health (MOH) areas were considered for the socio-economic survey.

### Selection of study population

An analytical cross-sectional survey was conducted from February to April 2017. The five most high-risk MOH areas that have reported the highest number of dengue cases during 2010–2016, were selected as the study areas in each district. A sample of 200 dengue impacted households were selected along with another 200 non-dengue reported households from a single MOH area (400 households from a single MOH area), on a systematic random basis following Krejcie & Morgan [16]. Cumulatively, 2000 dengue-impacted households and non-dengue households (with a ratio of 1:1) were used as the total sample from each district (400 × 5). During the calculation of sample size, it was assumed that the marginal error is 3.5% and population proportion is 0.5, while the actual population size of the whole Kandy and Colombo districts were taken as 1,369,899 and 2,309,809, respectively.

Households, from which the residents were not willing to cooperate in the study due to one or more reasons such as religious beliefs, absence of a household head or their opinion that it is not worthwhile participating in our survey, were not considered for the survey. On such occasions, the sample size was achieved by randomly selecting new households with consent to participate for the study.

### Data collection

The demographic, socio-economic and living standard related factors, along with knowledge, attitudes and practices (KAPs) of the selected households were obtained by using an interviewer-administered questionnaire prepared in three local languages (Sinhala, English and Tamil). The selected household heads were interviewed by a group of trained interviewers. The household head was defined as the person who is perceived by the household members to be the primary decision maker in the family and the household was defined as individuals living together and taking meals from a common cooking facility [14]. In the absence of a household head, a responsible adult above 18 years, appointed by the family, was considered for the study.

The questionnaire covered the following areas: (i) demographic information (age and gender of the household head, number of family members, monthly income of the family and number of years residing within the relevant MOH); (ii) living standards (size of the homestead, vegetation coverage, temporary or permanent nature of the household, status of the household, accessibility conditions, surrounding land use types, number of rooms in the house, roofing, drinking water source and sanitary conditions); (iii) knowledge about dengue vectors, symptoms of dengue, vector ecology and transmission modes; (iv) attitude towards dengue and dengue management approaches; and (v) preventive practices against dengue (source separation of solid waste, waste disposal methods, maintaining a clean environment, home gardening practices and composting, covering of water storage tanks, cleaning and proper maintenance of roof gutters, use of mosquito repellents and insecticides etc.).

### Data interpretation and statistical analysis

All collected data were double-checked and verified on the same day for completeness and consistency. The data were then entered into Microsoft Access® 2007 data sheets, while adhering to quality controlling procedures by trained personnel. The accuracy of data was routinely checked by cross tabulations and logical checks. Discrepant data were checked against original data forms and any mistakes were promptly corrected.

Chi-square test of independence was used for the statistical comparison of significance among study populations in terms of epidemiological, demographic, socio-economic and KAP factors [17, 18]. The proportions of demographic, epidemiological and socio-economic characteristics of the study populations were subjected to a cluster analysis (based on Euclidean distance) [19] followed by an analysis of similarities (ANOSIM) (i.e. a non-parametric analogue of MANOVA) [20], after square-root transformation. Furthermore, multi-dimensional scaling (MDS) analysis in Plymouth Routines in Multivariate Ecological Research version 6 (PRIMER 6) was utilized for the visual representation and comparison of the study samples in terms of overall socio-economic characterization [19].

## Results

### Demographic and socio-economic factors

The present study surveyed 4000 households as test (*n* = 2000) and control (*n* = 2000) groups in the two studied districts. The majority of the reported dengue patients from the households in test groups (dengue impacted), were males in both Kandy (60.8%) and Colombo (58.63%) districts. The age group of 15–35 years was predominant (Colombo, 42.2%; Kandy, 48.9%) among dengue patients. People > 55 years of age were the least susceptible group for dengue infection. The number of patients with a total monthly income > 30,000 LKR (1 USD = 159.81 LKR as of 5th of August 2018) was low in Colombo (29.0%) and Kandy (22.0%) districts with respect to the control population (Table 1). The average family size of > 7 members in the test population was low, compared to the control group. In both districts, all basic demographic factors denoted significant differences (Table 1) among the control and dengue patient populations.

**Table 1:** Summarized percentages for demographic and socio-economic characteristics of the study populations from the districts of Colombo and Kandy

Factor		Kandy (%)			Colombo (%)		
		Control *n* (%)	Positive *n* (%)	*P*-value^a^	Control *n* (%)	Positive *n* (%)	*P-*value^a^
Age in years	0–15	15 (1.50)	355 (35.5)	0.04*	18 (1.8)	207 (20.7)	0.03*
	15–35	277 (27.7)	422 (42.2)		302 (30.2)	488 (48.8)	
	35–55	495 (49.5)	155 (15.5)		517 (51.7)	230 (23.0)	
	> 55	213 (21.3)	66 (6.6)		162 (16.2)	75 (7.5)	
Sex	Male	903 (90.30)	608 (60.8)	0.01*	883 (88.3)	586 (58.6)	0.03*
	Female	970 (9.70)	392 (39.2)		117 (11.7)	414 (41.4)	
Family size	1–3	165 (16.5)	316 (31.6)	0.04*	247 (24.7)	396 (39.6)	0.03*
	4–6	520 (52.0)	504 (50.4)		458 (45.8)	498 (49.8)	
	> 7	315 (31.5)	180 (18.0)		295 (29.5)	106 (10.6)	
No. of years residing within the relevant MOH	< 5	146 (14.6)	302 (30.2)	0.02*	148 (14.8)	300 (30.0)	0.01*
	6–25	269 (26.9)	441 (44.1)		242 (24.2)	492 (49.2)	
	26–50	394 (39.4)	166 (16.6)		412 (41.2)	152 (15.2)	
	> 50	191 (19.1)	91 (9.1)		196 (19.6)	55 (5.5)	
Total monthly income in LKR × 10^3b^	< 5	3 (0.3)	4 (0.4)	0.02*	0 (0)	5 (0.5)	0.008**
	5–10	20 (2.0)	46 (4.6)		0 (0)	35 (3.5)	
	10–20	55 (5.5)	258 (25.8)		50 (5.0)	395 (39.5)	
	20–30	605 (60.5)	472 (47.2)		266 (26.6)	275 (27.5)	
	> 30	317 (31.7)	220 (22.0)		684 (68.4)	290 (29.0)	

^a^All *P*-values are based on a Chi-square test for independence analysis of numbers in dengue- and non-dengue patient populations (in respective districts, separately)

^b^1 USD = 159.81 LKR

*P < 0.05, **P < 0. 01

### Living standards of the community

#### House condition, infrastructure and surroundings

The land extent of the homesteads differed significantly among test and control groups in both Colombo (*χ*^2^ = 11.667, *df* = 4, *P* = 0.02) and Kandy (*χ*^2^ = 10.712, *df* = 4, *P* = 0.03). Homesteads with a land extent of less than 5 perches were higher in the dengue impacted groups in Kandy (29.5%) and Colombo (35.5%), than in the respective control groups (Table 2). The number of dengue impacted households with more than 1 floor (but less than 5 floors) was higher than the control group in the districts of Colombo (13.8%) and Kandy (14.2%). The house condition was associated with the occurrence of dengue (Table 2). Houses with plastered cement walls and roofing with tiles or asbestos sheets were considered as “Good”, while houses with un-plastered brick walls with tiled, asbestos or incomplete roofing (concrete slab) conditions were considered as “Moderate”. Houses other than these types were considered as “Poor” houses [14]. Even though the majority (> 60%) of households in all the study groups belonged to the “Moderate” category, the proportion of houses that fell into “Poor” category was significantly higher in the test group of Colombo (29.4%) with respect to the control (*χ*^2^ = 6.438, *df* = 2, *P* = 0.04) although it was not significant (*χ*^2^ = 4.816, *df* = 2, *P* = 0.09) in Kandy (Table 2).

**Table 2:** Summarized percentages for household characteristics of the study populations from the districts of Colombo and Kandy

Factor		Kandy			Colombo		
		Control *n* (%)	Positive *n* (%)	*P*-value^a^	Control *n* (%)	Positive *n* (%)	*P-*value^a^
Accessibility	Main road	88 (8.8)	126 (12.6)	0.30	135 (13.5)	138 (13.8)	0.42
	Medium/small road	867 (86.7)	823 (82.3)		825 (82.5)	810 (81.0)	
	Foot path/no road	45 (4.5)	51 (5.1)		40 (4.0)	52 (5.2)	
Size of the homestead (perch)	< 5	175 (17.5)	295 (29.5)	0.02*	208 (20.8)	355 (35.5)	0.03*
	6–10	275 (27.5)	301 (30.1)		295 (29.5)	312 (31.2)	
	11–25	403 (40.3)	298 (29.8)		387 (38.7)	253 (25.3)	
	26–50	93 (9.3)	61 (6.1)		64 (6.4)	43 (4.3)	
	> 50	54 (5.4)	45 (4.5)		46 (4.6)	37 (3.7)	
Temporary or permanent nature of the human dwelling	Permanent	974 (97.4)	935 (93.5)	0.12	967 (96.7)	945 (94.5)	0.16
	Temporary	26 (2.6)	65 (6.5)		33 (3.3)	55 (5.5)	
No. of houses in the land plot	1	911 (91.1)	942 (94.2)	0.17	975 (97.5)	886 (88.6)	0.08
	2–3	87 (8.7)	58 (5.8)		25 (2.5)	114 (11.4)	
	> 4	2 (0.2)	0 (0)		0 (0)	0 (0)	
Type of household	Individual house	952 (95.2)	858 (85.8)	0.11	950 (95.0)	862 (86.2)	0.14
	< 5 floors	48 (4.8)	142 (14.2)		50 (5.0)	138 (13.8)	
	> 5 floors	0 (0)	0 (0)		0 (0)	0 (0)	
	Other	0 (0)	0 (0)		0 (0)	0 (0)	
Residential function of the household	Residential only	947 (94.7)	898 (89.8)	0.15	950 (95.0)	872 (87.2)	0.14
	Residential and commercial	40 (4.0)	48 (4.8)		38 (3.8)	41 (4.1)	
	Small industry	13 (1.3)	54 (5.4)		12 (1.2)	67 (6.7)	
	Commercial only	0 (0)	0 (0)		0 (0)	20 (2.0)	
Status of the household	Good	281 (28.1)	164 (16.4)	0.09	132 (13.2)	45 (4.5)	0.04*
	Moderate	613 (61.3)	606 (60.6)		796 (79.6)	661 (66.1)	
	Poor	106 (10.6)	230 (23.0)		72 (7.2)	294 (29.4)	
Number of rooms in the house	1	52 (5.2)	26 (2.6)	0.17	7 (0.7)	32 (3.2)	0.19
	2–3	903 (90.3)	862 (86.2)		916 (91.6)	939 (93.9)	
	4–6	41 (4.1)	100 (10.0)		61 (6.1)	26 (2.6)	
	> 6	4 (0.4)	12 (1.2)		16 (1.6)	3 (0.3)	
Vegetation coverage	Grass	385 (38.5)	627 (62.7)	0.03*	382 (38.2)	620 (62.0)	0.04*
	Bushes	397 (39.7)	617 (61.7)		430 (43.0)	646 (64.6)	
	Small trees	469 (46.9)	616 (61.6)		485 (48.5)	425 (42.5)	
	Large trees	468 (46.8)	479 (47.9)		366 (36.6)	210 (21.0)	
Surrounding land-use practices in the neighborhood	Agricultural areas	125 (12.5)	72 (7.2)	0.03*	78 (7.8)	55 (5.5)	0.02*
	Water bodies	17 (1.7)	83 (8.3)		21 (2.1)	103 (10.3)	
	Built-up	620 (62.0)	892 (89.2)		655 (65.5)	931 (93.1)	
	Marshy	153 (15.3)	326 (32.6)		52 (5.2)	241 (24.1)	
	Abundant	102 (10.2)	207 (20.7)		97 (9.7)	197 (19.7)	
	Other	33 (3.3)	41 (4.1)		28 (2.8)	45 (4.5)	
Toilets	Separate (outside)	699 (69.9)	775 (77.5)	0.31	431 (43.1)	614 (61.4)	0.04*
	Attached	181 (18.1)	118 (11.8)		324 (32.4)	225 (22.5)	
	Both	109 (10.9)	87 (8.7)		245 (24.5)	116 (11.6)	
	None	11 (1.1)	20 (2.0)		0 (0)	45 (4.5)	
Water source	Well	8 (0.8)	6 (0.6)	0.14	85 (8.5)	55 (5.5)	0.11
	Tube-well	27 (2.7)	8 (0.8)		0 (0)	0 (0)	
	Pipe	964 (96.4)	961 (96.1)		890 (89.0)	912 (91.2)	
	Other	1 (0.1)	25 (2.5)		25 (2.5)	33 (3.3)	
Protection of tank, *n* (%)	Fully covered	938 (93.8)	811 (81.1)	0.06	933 (93.3)	635 (63.5)	0.03*
	Partially covered	40 (4.0)	114 (11.4)		22 (2.2)	185 (18.5)	
	Not covered	22 (2.2)	75 (7.5)		45 (4.5)	180 (18.0)	
Roofing	Concrete	290 (29.0)	353 (35.3)	0.04*	257 (25.7)	382 (38.2)	0.03*
	Roof tiles	392 (39.2)	313 (31.3)		412 (41.2)	373 (37.3)	
	Asbestos	673 (67.3)	837 (83.7)		708 (70.8)	845 (84.5)	
	Metal sheets	259 (25.9)	482 (48.2)		242 (24.2)	450 (45.0)	
	Other	49 (4.9)	214 (21.4)		53.8 (5.38)	171 (17.1)	

^a^All *P*-values are based on a Chi-square test for independence analysis of numbers in dengue- and non-dengue patient populations (in respective districts, separately)

*P < 0.05

The major roofing material was asbestos sheets in Kandy (83.7%) and in Colombo (84.5%), followed by metal sheets, concrete and roof tiles. Most of the households did not have roof gutters, while many had been removed due to the earlier prevalence of dengue epidemics, particularly in the test groups of both Colombo (*n* = 485) and Kandy (*n* = 415).

Vegetation coverage at the homesteads in test and control clusters also varied significantly in both districts. The relative coverage of grass and bushes was higher in dengue impacted households of both Colombo (*χ*^2^ = 8.311, *df* = 3, *P* = 0.04) and Kandy (*χ*^2^ = 8.151, *df* = 3, *P* = 0.03) districts. Homesteads of dengue patients in Kandy and Colombo were surrounded by built-up environments followed by marshy and abandoned lands (Table 2). Medium and small roads were the dominant access routes in all the groups, while the majority of the households were permanent and mainly utilized for residential purposes.

The location of toilets and access to and storage of water in the households was an important determinant in the occurrence of dengue. Although pipe-borne water was the major source of water in the study groups of the Kandy and Colombo districts, the protection of the stored water tanks differed significantly (*χ*^2^ = 7.013, *df* = 3, *P* = 0.03) between the dengue positive households and the non-dengue reported households in Colombo. Households with partially protected (18.5%) or non-protected (18%) water storage tanks were more vulnerable for the breeding of dengue vectors (Table 2).

### Knowledge, attitudes and practices on dengue

#### Knowledge on dengue

The status of knowledge within the study groups on different aspects of dengue relevant to infection, symptoms, transmission and ecology of vectors was assessed. The control group in both districts had a better awareness on dengue transmission than the test group. However, the possibility of multiple infections of dengue was a lesser known fact in the control group in Kandy (69.8%), while the test group had a better awareness level (82.5%). The test groups in Kandy (79.2%) and Colombo (80.8%) districts also had a better awareness level on symptoms of dengue with reference to control groups in the present study. Detailed symptoms such as muscular pain and occurrence of a rash were less familiar among the individuals of the control cluster.

However, the control groups were more aware of the biting habit of vectors and morphology of *Aedes* than the test group. All the study populations had an acceptable level of awareness on vector breeding, where the knowledge level of control groups (88.3% and 83.8% for Colombo and Kandy, respectively) was better than the test groups. Only a low proportion of the community (particularly the dengue patients) were aware of the ability of dengue vectors to breed in water retained in the leaf axils of plants and water retention trays in air-conditioners and refrigerators (Table 3). The average knowledge level of the control and test clusters under different categories varied significantly (*χ*^2^ = 15.42, *df* = 4, *P* = 0.04) in the district of Colombo (Table 3). Although a similar trend was observed in Kandy, the results were not statistically significant (*χ*^2^ = 5.94, *df* = 4, *P* = 0.20).

**Table 3:** Summarized knowledge characteristics of the study populations in the districts of Colombo and Kandy

Factor	Kandy (%)		Colombo (%)	
	Control *n* (%)	Positive *n* (%)	Control *n* (%)	Positive *n* (%)
Transmission of dengue				
Dengue is caused by a virus	874 (87.4)	650 (65.0)	848 (84.8)	578 (57.8)
Dengue virus has four serotypes	685 (68.5)	549 (54.9)	794 (79.4)	615 (61.5)
A person is vulnerable to dengue more than once	698 (69.8)	825 (82.5)	785 (78.5)	495 (49.5)
Dengue is transmitted by a mosquito	1000 (100)	1000 (100)	1000 (100)	1000 (100)
*Ae. aegypti* and *Ae. albopictus* are the vectors of dengue	945 (94.5)	735 (73.5)	845 (84.5)	687 (68.7)
Bites of infected mosquitoes may cause dengue to a healthy person	845 (84.5)	885 (88.5)	925 (92.5)	907 (90.7)
Symptoms of dengue				
Fever	1000 (100)	1000 (100)	1000 (100)	1000 (100)
Joint pains	755 (75.5)	879 (87.9)	678 (67.8)	945 (94.5)
Rash	648 (64.8)	818 (81.8)	547 (54.7)	857 (85.7)
Headache	725 (72.5)	947 (94.7)	697 (69.7)	925 (92.5)
Muscular pain	588 (58.8)	895 (89.5)	512 (51.2)	945 (94.5)
Nausea/vomiting	614 (61.4)	754 (75.4)	567 (56.7)	785 (78.5)
Other	185 (18.5)	248 (24.8)	128 (12.8)	199 (19.9)
Most frequent bite time				
At night	118 (11.8)	278 (27.8)	58 (5.8)	187 (18.7)
Daytime	713 (71.3)	542 (54.2)	787 (78.7)	489 (48.9)
Both day and night	169 (16.9)	180 (18.0)	155 (15.5)	324 (32.4)
Vector morphology				
Black in color	874 (87.4)	678 (67.8)	905 (90.5)	532 (53.2)
Have white spots on their legs	657 (65.7)	452 (45.2)	725 (72.5)	417 (41.7)
Slightly brownish in color	126 (12.6)	322 (32.2)	95 (9.5)	458 (45.8)
Vector breeding				
Breed in standing water	957 (95.7)	905 (90.5)	980 (98.0)	885 (88.5)
Breed in clean water	974 (97.4)	955 (95.5)	984 (98.4)	917 (91.7)
Breed in leaf axils and plant surfaces	675 (67.5)	412 (41.2)	784 (78.4)	457 (45.7)
Breed in water retention tanks in A\C machines and refrigerators	745 (74.5)	510 (51.0)	782 (78.2)	528 (52.8)
Summarized knowledge scores				
Transmission of dengue	841 (84.1)	774 (77.4)	866 (86.6)	714 (71.4)
Symptoms of dengue	645 (64.5)	792 (79.2)	589 (58.9)	808 (80.8)
Biting behaviour	713 (71.3)	542 (54.2)	787 (78.7)	489 (48.9)
Vector morphology	766 (76.6)	565 (56.5)	815 (81.5)	475 (47.5)
Vector breeding	838 (83.8)	696 (69.6)	883 (88.3)	697 (69.7)
Chi-square statistics	*χ*^2^ = 5.94, *P* = 0.20^a^		*χ*^2^ = 15.42, *P* = 0.04^b^*	

^a^*P-*value of the Chi-square test for independence analysis of numbers in dengue and non-dengue patient populations in Kandy with respect to summarized knowledge scores

^b^*P-*value of the Chi-square test for independence analysis of numbers in dengue and non-dengue patient populations in Colombo with respect to summarized knowledge scores

*P < 0.05

The majority of the control (43.5%) and test (46.9%) clusters in Kandy were unaware that they were residing in a high dengue risk area with frequent dengue cases. In Colombo, the control group had a significantly higher (*χ*^2^ = 6.438, *df* = 2, *P* = 0.04) level of familiarity of the current status of dengue in their area of residence (Table 4).

**Table 4:** Summarized attitudes of the study populations towards dengue

Factor		Kandy (%)			Colombo (%)		
		Control *n* (%)	Positive *n* (%)	*P*-value (*χ*^2^)^a^	Control *n* (%)	Positive *n* (%)	*P*-value (χ^2^)
Case frequency	Frequent	533 (53.3)	469(46.9)	0.02^*^	847 (84.7)	685 (68.5)	0.04^*^
	Occasionally	435 (43.5)	526 (52.6)		153 (15.3)	315 (31.5)	
	None	12 (1.2)	5 (0.5)		0 (0)	0 (0)	
Need more awareness	Yes	623 (62.3)	779 (77.9)	0.13	825 (82.5)	652 (65.2)	0.16
	No	377 (37.7)	221 (22.1)		175 (17.5)	348 (34.8)	
If yes, in which aspects	Symptoms and treatments of DHF	686 (68.6)	468 (46.8)	0.03^*^	825 (82.5)	558 (55.8)	0.02^*^
	Controlling	651 (65.1)	539 (53.9)		785 (78.5)	395 (39.5)	
	Solid Waste Management (SWM)	447 (44.7)	299 (29.9)		645 (64.5)	387 (38.7)	
Attitudes on community based vector management	Yes	757 (75.7)	576 (57.6)	0.03^*^	855 (85.5)	645 (64.5)	0.04^*^
	No	243 (24.3)	424 (42.4)		145 (14.5)	355 (35.5)	
Have there been adequate steps taken to control dengue?	Yes	289 (28.9)	141 (14.1)	0.03*	455 (45.5)	205 (20.5)	0.01*
	No	711 (71.1)	859 (85.9)		545 (54.5)	795 (79.5)	
The responsible party for management of dengue	Government	659 (65.9)	851 (85.1)	0.04*	750 (75.0)	897 (89.7)	0.03*
	Non-governmental organizations (NGO)	120 (12.0)	48 (4.8)		355 (35.5)	188 (18.8)	
	Community	255 (25.5)	165 (16.5)		71 (7.10)	11 (1.1)	
Role of the Public Health Inspector (PHI)	Excellent	291 (29.1)	65 (6.5)	0.005**	355 (35.5)	205 (20.5)	0.04*
	Satisfactory	298 (29.8)	42 (4.2)		225 (22.5)	170 (17.0)	
	Moderate	339 (33.9)	153 (15.3)		282 (28.2)	305 (30.5)	
	Unsatisfactory	72 (7.2)	740 (74.0)		138 (13.8)	320 (32.0)	

^a^All *P*-values are based on a Chi-square test for independence analysis of numbers in dengue- and non-dengue patient populations (in respective districts, separately)

*P < 0.05, **P < 0.01

#### Attitudes on dengue infection and vector control

A high percentage of the participants were willing to further improve their awareness and knowledge on dengue, particularly on the aspects of symptoms and treatments for dengue and controlling of dengue vector breeding. In both districts, the control groups had a significantly higher need for further knowledge (*χ*^2^ = 7.824, *df* = 2, *P* = 0.02 and *χ*^2^ = 7.013, *df* = 2, *P* = 0.03, for Colombo and Kandy, respectively) on these aspects than the participants from dengue impacted households (Table 4).

More than 70% of the participants of all study groups, except for the control population of Colombo (54.5%), were not satisfied about the existing vector control programmes (Table 4). However, the individuals in control groups of Colombo (*χ*^2^ = 6.635, *df* = 1, *P* = 0.01) and Kandy (*χ*^2^ = 4.709, *df* = 1, *P* = 0.03) indicated a relatively higher level of satisfaction about the control efforts implemented by the vector controlling entities. A higher proportion of all study groups believed that government is the major responsible body, which should be directly involved in the management of dengue incidences and epidemics. In Kandy, the community was recognized as the second responsible party followed by non-governmental organizations (NGOs), while the opposite was observed from the study in Colombo (Table 4). The majority of participants were willing to contribute to any community-based vector management strategy for dengue in both districts. However, a significantly higher percentage of the control groups were volunteering to involve in community-based dengue management activities in the districts of Colombo (*χ*^2^ = 4.217, *df* = 1, *P* = 0.04) and Kandy (*χ*^2^ = 4.709, *df* = 2, *P* = 0.03).

The test groups in the districts of Colombo (32%) and Kandy (74%) were not satisfied regarding the services provided by field-based health workers in terms of follow-up action and vector control interventions. Except for a need of more awareness, rest of the parameters in the questionnaire (Table 4) differed significantly between the test and control groups.

### Practices related to dengue control

#### Waste disposal and management practices

More than 70% of the households maintained a clean environment around house premises. The control groups in Colombo (*n* = 285) and Kandy (*n* = 177) practiced composting or home gardening, more frequently than the test groups. Limitations in time/space were the major contributors for those who were not practicing composting or home gardening. Disposing waste *via* collection trucks of local government agencies (with a frequency of < 7 days) was the most practiced method of waste disposal, followed by burning and disposing into a garbage pit. Interestingly, 22.7% of the control households in Colombo practiced composting. Although most did not practice source separation of solid waste, 48.5% of the control households in Colombo practiced source separation, prior disposal of solid waste.

However, the fraction of respondents practicing proper disposal of solid waste, organizing “Shramadana” (cleaning campaigns) to clean the surrounding and clearing bushes and other vegetation were relatively higher among both dengue-free control groups, which may be the reason for the significant difference between the practices against vector breeding among the control and patient groups in Colombo (*χ*^2^ = 16.622, *df* = 7, *P* = 0.02) and Kandy (*χ*^2^ = 18.475, *df* = 7, *P* = 0.01) districts (Table 5).

**Table 5:** Practices of the study populations towards dengue

Factors		Kandy (%)			Colombo (%)		
		Control *n* (%)	Positive *n* (%)	*P-*value (*χ*^2^)^a^	Control *n* (%)	Positive *n* (%)	*P-*value (*χ*^2^)^a^
Are the premises clean?	Yes	911 (91.1)	847 (84.7)	0.19	875 (87.5)	724 (72.4)	0.15
	No	89 (8.9)	153 (15.3)		125 (12.5)	276 (27.6)	
Practicing of composting/home gardening	Yes	178 (17.8)	45 (4.5)	0.07	285 (28.5)	79 (7.9)	0.04*
	No	822 (82.2)	955 (95.5)		715 (71.5)	921 (92.1)	
If no, due to	Limitations in time	472 (47.2)	679 (67.9)	0.04*	784 (78.4)	857 (85.7)	0.63
	Limited space	572 (57.2)	744 (74.4)		627 (62.7)	785 (78.5)	
	Limited labor	309 (30.9)	482 (48.2)		475 (47.5)	358 (35.8)	
Waste disposal frequency	Daily	133 (13.3)	57 (5.7)	0.12	247 (24.7)	125 (12.5)	0.04*
	< 7 days	856 (85.6)	920 (92.0)		753 (75.3)	845 (84.5)	
	> 7 days	11 (1.1)	23 (2.3)		0 (0)	30 (3)	
Waste disposal method	Garbage pit	152 (15.2)	277 (27.7)	0.13	58 (5.8)	35 (3.5)	0.17
	Collected by Municipal Council	755 (75.5)	702 (70.2)		855 (85.5)	757 (75.7)	
	At the roadside	0 (0.0)	0 (0.0)		55 (5.5)	125 (12.5)	
	Open ground dumping	0 (0.0)	0 (0.0)		25 (2.5)	75 (7.5)	
	Composting	95 (9.5)	12 (1.2)		227 (22.7)	55 (5.5)	
	Burning	325 (32.5)	390 (39.0)		185 (18.5)	157 (15.7)	
Limitations in the service provided by Pradeshiya Sabha (PS) for waste disposal	Once in 2 weeks	7 (0.7)	45 (4.5)	0.04*	8 (0.8)	36 (3.6)	0.02*
	Once per week	816 (81.6)	746 (74.6)		888 (88.8)	777 (77.7)	
	Irregular collection date	611 (61.1)	778 (77.8)		558 (55.8)	752 (75.2)	
	Use of no alarming sound to inform the residents that they are visiting the area	264 (26.4)	392 (39.2)		185 (18.5)	245 (24.5)	
	Do not reach the road or house	258 (25.8)	700 (70.0)		285 (28.5)	780 (78.0)	
	Rejection of items	862 (86.2)	922 (92.2)		405 (40.5)	620 (62.0)	
Practicing of source separation of solid waste	Yes	289 (28.9)	202 (20.2)	0.16	485 (48.5)	322 (32.2)	0.04*
	No	711 (71.1)	798 (79.8)		515 (51.5)	677 (67.7)	
Prevention methods against mosquito bites	Use of screen	244 (24.40)	53 (5.30)	0.02*	162 (16.2)	45 (4.5)	0.03*
	Closing of windows	320 (32.0)	125 (12.5)		355 (35.5)	125 (12.5)	
	Coils/creation of smoke	723 (72.3)	634 (63.4)		782 (78.2)	845 (84.5)	
	Nets	628 (62.8)	257 (25.7)		722 (72.2)	325 (32.5)	
	Fans	274 (27.4)	331 (33.1)		575 (57.5)	285 (28.5)	
	Other	381 (38.1)	203 (20.3)		145 (14.5)	98 (9.8)	
	None	16 (1.6)	17 (1.7)		0 (0)	5 (0.5)	
Functioning of roof gutters	Functioning	416 (41.6)	213 (21.3)	0.04*	400 (40.0)	195 (19.5)	0.04*
	Blocked	6 (0.6)	23 (2.3)		2 (0.2)	21 (2.1)	
	None	578 (57.8)	764 (76.4)		597 (59.7)	783 (78.3)	
	Removed after severe epidemics	385 (38.5)	415 (41.5)		385 (38.5)	485 (48.5)	
Practices against prevention of mosquito breeding	Eliminate potential breeding sites	645 (64.5)	353 (35.3)	0.01*	563 (56.3)	445 (44.50)	0.02*
	Cleaning the garden twice per week	565 (56.5)	185 (18.5)		455 (45.5)	225 (22.50)	
	Adding fish to ponds	453 (45.3)	234 (23.4)		782 (78.2)	845 (84.5)	
	Proper disposal of solid waste	658 (65.8)	357 (35.7)		722 (72.2)	325 (32.5)	
	Organizing “Shramadana” to clean the surroundings	274 (27.4)	81 (8.1)		205 (20.5)	75 (7.5)	
	Covering water containers and tanks	938 (93.8)	811 (81.1)		933 (93.3)	630 (63.0)	
	Clearing bushes and other vegetation	426 (42.6)	155 (15.5)		245 (24.5)	55 (5.5)	

^a^All *P*-values are based on a Chi-square test for independence analysis of numbers in dengue- and non-dengue patient populations (in respective districts, separately)

*P < 0.05

#### Prevention of vector breeding and biting

Covering water storage containers/tanks and eliminating potential breeding sites of vector mosquitoes were the most common practices in both districts. The use of mosquito coils and creation of smoke were the most practiced measures to prevent mosquito bites followed by net use and closing windows (Table 5). Mosquito bite prevention methods among test and control groups differed significantly in both districts (*χ*^2^ = 15.509, *df* = 7, *P* = 0.03 and *χ*^2^ = 16.622, *df* = 7, *P* = 0.02 for Colombo and Kandy, respectively). The use of mosquito coils, creation of smoke and use of fans remained as the most common methods of vector-human contact minimization in the test populations. On the other hand, apart from the above practices, the proportion of households that used screens for doors and windows, practice closing of windows at dawn and dusk and applied other methods such as mosquito repellents, etc. were high among the dengue non-impacted group (Table 5).

### Overall characterization of study populations

**Fig. 2:**
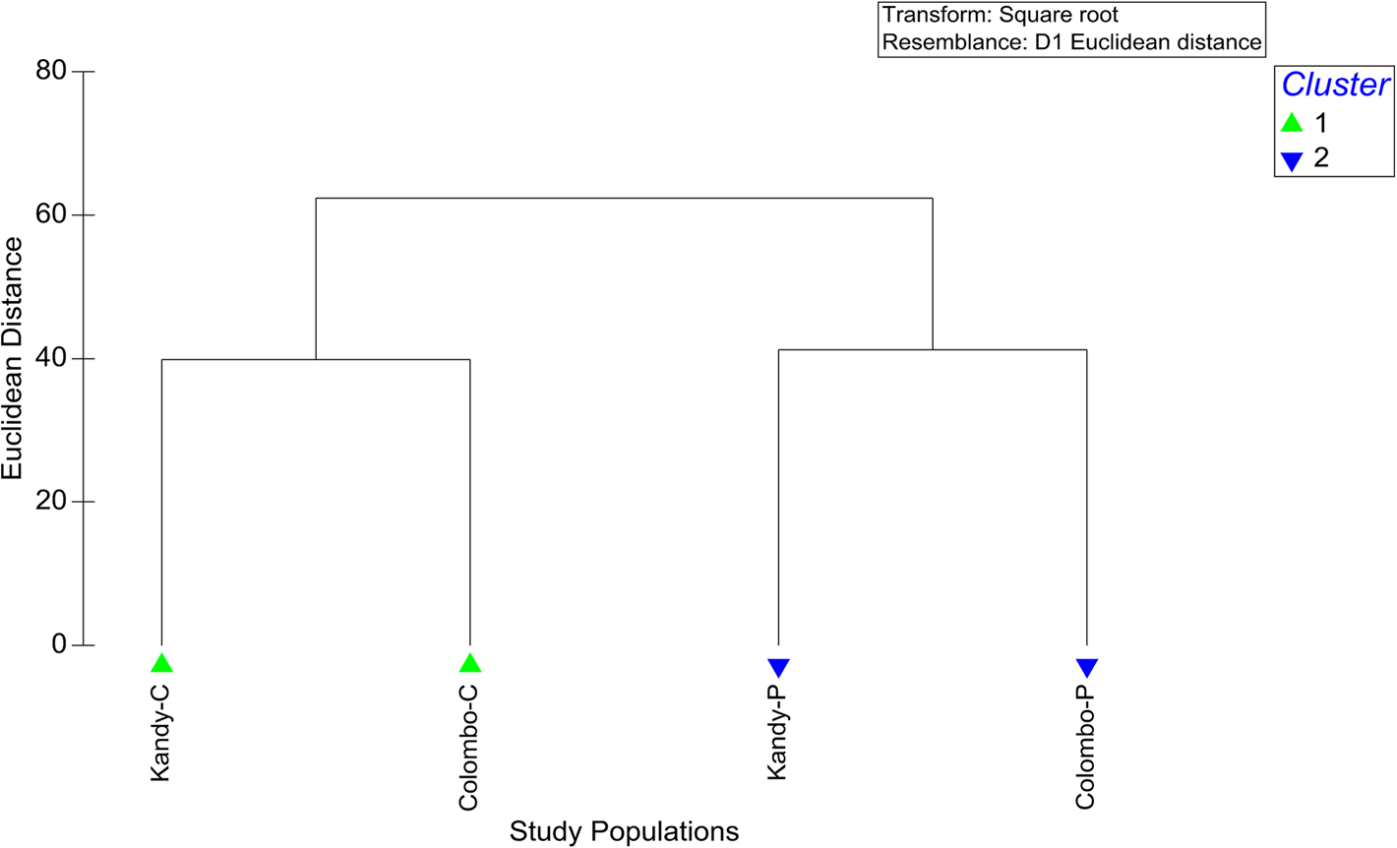
Dendrogram of the cluster analysis of the study populations in terms of the studied demographic, epidemiological and socio-economic characteristics. *Abbreviations*: Colombo-C, non-dengue patient group from Colombo; Colombo-P, dengue patient group from Colombo; Kandy-C, non-dengue patient group from Kandy; Kandy-P, dengue patient group from Kandy

**Fig. 3:**
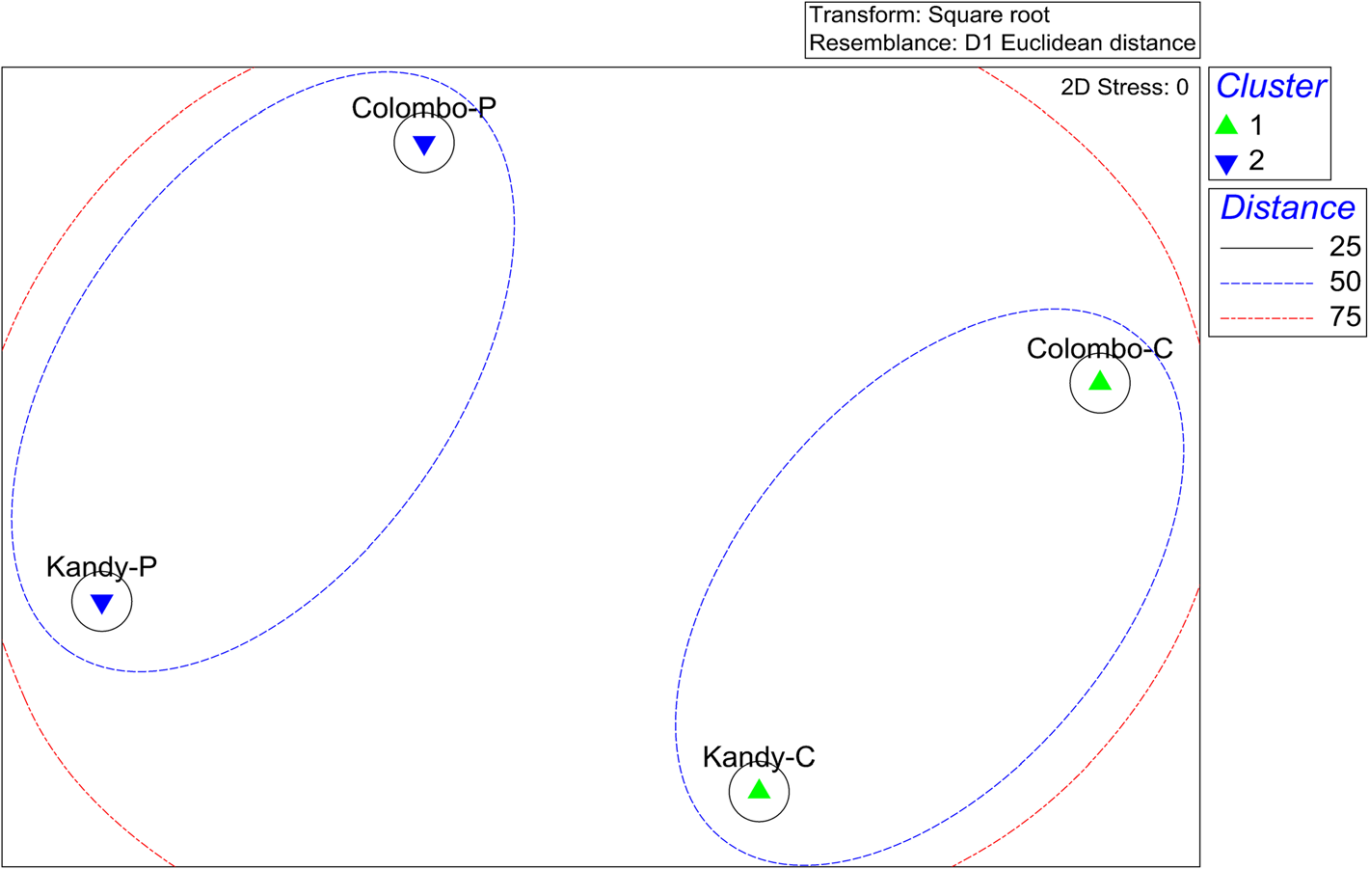
Multidimensional scaling (MDS) plot for the study populations in terms of overall demographic, KAPs, socio-economic and living standard related characteristics. *Abbreviations*: Colombo-C, non-dengue patient group from Colombo; Colombo-P, dengue patient group from Colombo; Kandy-C, non-dengue patient group from Kandy; Kandy-P, dengue patient group from Kandy

Although study groups were associated with two distinct geographical and urbanization levels, the four study groups clustered into two major clusters at a Euclidean distance of 40 as dengue-impacted (test) and dengue non-impacted (control) groups based on the overall demographic, KAPs, socio-economic and living standard related characteristics (Fig. 2). The analysis of similarities (ANOSIM) further confirmed the formation of the above clusters by yielding a Global *R* value of 0.99 (*P* = 0.033). The multidimensional scaling (MDS) plot also resulted in the same observation (Fig. 3).

## Discussion

Since the first incidence of a dengue outbreak in 1989, a series of outbreaks has lifted dengue up to the level of a major health issue presently faced by Sri Lanka, along with expansions in the geographical range. The year 2017, when the highest number of dengue cases within the country was reported, has become a crucial turning point for the vector controlling entities in Sri Lanka [5, 14]. However, detailed, in-depth studies on the socio-economic, living standards, and knowledge, attitude and practice aspects based risk characterization of dengue are still limited in Sri Lanka [14]. Therefore, the present study was conducted to fill the above gap, while assisting the management of dengue epidemics through proper community mobilization.

### Demographic factors

The importance of demographic and socio-economic aspects in dengue control programmes, especially during disease epidemic episodes, has been progressively recognized by many countries [21–23]. In both dengue-positive groups of Colombo and Kandy districts, males belonging to the 15–35 age category who usually spend a notable time in public environments (school, tuition classes, work places and bus stands etc.) mainly for occupation or education purposes, have been recognized as the most susceptible category for dengue. Therefore, they are more exposed to the contact with *Aedes* vectors, which may be responsible for their high disease susceptibility [24].

Human movement, especially between urban/semi-urban and rural environments, has been recognized to significantly contribute to the increased transmission of dengue, confirming the above deduction [25, 26]. Findings of Udayanga et al. [14] and Nadeeka et al. [27] have also reported a similar trend regarding the susceptibility of younger age groups, while several studies conducted in other countries such as Brazil [28], Puerto Rico [29], Singapore [30] and Thailand [31], have reported a reverse trend. The presence of a high number of family members was a contributing factor for low transmission of dengue, especially within the dengue-free populations. According to Alobuia et al. [32], the existence of a high number of family members in a household has been recognized as imposing high responsibility on parents or guardians, thereby encouraging them to maintain a clean and safe environment to prevent their family members becoming infective.

Families with higher income levels were having lower rates of dengue incidence, as their economic strength and education level would enable them to take effective preventive actions against dengue [33]. However, it was interesting to note that a study conducted in urban areas of Thailand, reported a higher dengue incidence rate among people with secondary and higher degrees of education than with a lower level of education [31]. High dengue prevalence rates among residents with a relatively shorter residence time in a neighborhood was another special feature highlighted by the current study, which had already been reported from several other studies [31].

### House conditions and infrastructural characteristics

Dominant characteristics of the living environment, especially the degree of urbanization, house density and surrounding land use practices, often influence the vulnerability to dengue outbreaks [14, 23, 27, 31]. As suggested by Alobuia et al. [32], occupants of moderate or smaller households have a relatively lower possibility of contacting dengue, since they tend to initiate necessary prevention and protective practices while maintaining a cleaner environment, when compared to the families living in relatively larger households. The number of dengue positive households with more than 1 floor (< 5 floors) were relatively higher than that of the control group in both districts of Colombo and Kandy. Hence, the apartment buildings could be a possible risk factor that support the transmission of dengue due to social negligence.

The relative coverage of grass and bushes (maintained as horticultural vegetation), were higher in households of dengue patients, which may provide ideal resting habitats for *Ae. albopictus* that remain as the secondary vector of dengue [34–36]. The high prevalence of built-up environment (urban environment) and marshy land has been found to be a critical risk factor associated with the incidence of dengue outbreaks in many countries including Malaysia [37, 38], Thailand [34] and Sri Lanka [14]. Such land use types may provide ideal breeding and resting grounds for *Ae. aegypti*, the primary vector for dengue with a high preference for urban settings. In particular, swamps and marshy land that may hold a shallow layer of standing freshwater provide ideal breeding grounds for *Aedes* mosquitoes, raising the risk associated with such surrounding land uses [34, 39]. Furthermore, majority of abandoned lands in two study areas were noted to be misused by the community, especially for improper disposal of solid waste, resulting in increased dengue vector populations [33].

Concrete, asbestos and metal sheets were the most preferred methods of roofing in all the studied communities. Such roofing materials, especially concrete and blocked roof gutters, have been identified as potential risk factors associated with dengue, especially during the rainy season by many studies conducted throughout the world [40–43]. However, the majority of the dengue recorded households claimed to remove roof gutters after an incidence of dengue, indicating the effectiveness of awareness programmes conducted within the high risk areas by different entities.

Almost all participants of the study heavily depended upon pipe-borne water, which is often associated with water storage tanks. It was noted that the relative percentage of partially covered or uncovered water storage facilities in both dengue patient groups was higher than the control groups; these facilities may provide stable breeding grounds for *Aedes* vectors increasing the possibility of dengue outbreaks [44, 45]. Furthermore, water storage and retention time period were relatively higher in dry periods in the country, favoring storage of excess water to cater to the day-to-day requirements, due to the limited supply of water. Such dry conditions may also be an indirect driving factor for the breeding of vectors as suggested by a recent study conducted in Australia [46]. Regarding toilet facilities, the majority of dengue patient households had separated toilets, mostly in outdoor settings. Such toilets may often contain water storage tanks or basins (especially in semi-urban or rural localities and in public toilets), which also could act as ideal breeding grounds for dengue vectors [45].

### Knowledge, attitudes and practices on dengue

High levels of knowledge on transmission, symptoms, patient care and prevention of vector breeding, have found to lower the risk of dengue incidence among communities from all over the world [13, 14, 47]. All the participants were aware of the fact that dengue is transmitted by the bites of infected mosquitoes and a large majority was even capable of naming the primary and secondary vectors of dengue. A study from Laos has also reported a similar situation whereby about 93% of the participants knew the name of the specific vector of dengue [48]. Furthermore, a notable fraction had the knowledge on the presence of four serotypes of dengue, even though some (especially the dengue patients from Colombo) were not aware of the fact that a single person is vulnerable to dengue more than once. The moderate to high literacy level in Colombo and Kandy (approximately 96.3% for Sri Lanka in 2015), and the continuous awareness programmes may be the contributing factors for maintaining higher levels of knowledge on dengue transmission [14]. A study conducted in Jamaica [47] also reported similar findings, while an opposite trend has been reported in Pakistan [48] and Nepal [13] whereby the respondents were only aware of the transmission by mosquitoes without any in-depth knowledge of the transmission.

Regarding the symptoms of dengue, all participants were capable of identifying lasting fever as the typical symptom along with headache (> 70%). However, a relatively higher percentage of dengue patients were capable of stating joint pains, rash, muscular pain and nausea/vomiting as other possible symptoms. A lower proportion identified other symptoms such as retro-orbital pain, abdominal pain and itching sensation. In general, a considerable amount of respondents were able to correctly identify typical symptoms of dengue, unlike several other studies conducted in India [49], Thailand [50], Laos [48], Nepal [13] and Jamaica [47]. However, dengue patient groups had a relatively higher level of knowledge on symptoms of dengue, than the participants of the control groups with no personal experiences of dengue in their households [13, 47]. The relatively acceptable level of knowledge on dengue symptoms may also be the outcome of awareness activities conducted within the relevant study areas. Such notable levels of knowledge on the symptoms may minimize the chance of patients confusing them with other general causes of fever such as influenza, typhoid, etc. and thereby enabling them to receive the required patient care from the health sector of Sri Lanka [47].

*Aedes* mosquitoes are known day-biters who prefer to engage in blood-feeding mainly during several hours after dawn and before dusk [13]. Unlike a study conducted in Jamaica, where only about 3% of respondents were aware of this biting behavior of dengue vectors [47], a notable fraction of Sri Lankan respondents was aware of the day-biting preference. However, the awareness level among dengue patient groups were low (especially in Colombo with only 48.9%), that may have caused their high vulnerability to dengue, since adequate preventive measures are not followed by them during the daytime. The situation may be more serious since they have not yet gathered the correct and important information about the disease even after the infection. In addition, the majority of the respondents were familiar with the basic facts of vector breeding such as the preference of vectors to breed in clean standing water and potential breeding sites in the household and premises that enable them to considerably reduce vector breeding at their premises [13]. Surprisingly, a majority of the control groups were familiar with the basic morphology of dengue vectors (presence of white spots on the legs and black-colored body), while only a lower fraction among the test groups were familiar with the above morphological features. A similar moderate level of knowledge on vector morphology has been reported from another study conducted in Saudi Arabia [51]. However, a previous study conducted in Kandy has reported a relatively lower level of awareness on the vector morphology [14]. Moderate to high levels of awareness on dengue was found among dengue patient and non-dengue patient groups in the present study. The efficacy of intensive awareness programmes conducted by different parties (government, NGO and other community based organizations) in improving the knowledge of community on different aspects of dengue could be the reason for the current knowledge level.

Most of participants in all four study groups knew that they were residing within high dengue risk areas. However, the percentage of dengue patients with the above understanding was relatively lower in both districts. More than three quarters of all four study groups desired to further improve their knowledge on numerous aspects of dengue, such as general transmission, symptoms and patient care of dengue, along with the control of vector breeding and vector contact. It is interesting to note that a considerable fraction is also willing to focus on solid waste management (SWM), which remains a key factor in dengue epidemic incidence. Many studies have highlighted the role played by solid waste management practices in governing dengue outbreaks [33, 52]. Therefore, the current desire of the community to further increase their knowledge is a positive indicator symbolizing that the general public is ready to take part in the process of dengue control through the reduction of vector breeding habitats.

The satisfaction of the community on the adequacy of steps taken by the government stakeholders in managing dengue was very poor (except for the control group of Colombo with 45.5% satisfaction). The limitations in the role played by field-based staff, who are responsible for the coordination and initiation of vector management activities at the ground level, may be a potential factor for such dissatisfaction [14]. Furthermore, the poor coordination between government entities and other stakeholders (including NGOs, the private sector and the community), outdated vector management strategies and local political conflicts, could also be listed as potential reasons for the low efficacy of government vector controlling activities.

Even though the government was named as the responsible party for the management of dengue, a considerable portion (approximately one quarter) understands that the community itself has a role to play in managing the dengue outbreaks. The highly positive attitudes on community-based vector management among study groups (especially in the control groups), bear evidence for the fact that local communities have also ascertained the potential risk of dengue and are ready to join hands with VCE to fight against it. The recent severe outbreaks of dengue might be the motivation factor for current attitudinal change of the people, which made them realize about their responsibilities in vector management. However, the current finding might also be partially influenced by the respondents trying to appear responsible in front of a stranger by providing socially desirable responses, without expressing their true self. Similar difficulties have also been reported in several other studies [13, 47].

Only a limited number of participants were practicing composting or home gardening, in all study populations, with a greater number contributing from the control groups. Even though government and private sector based agricultural entities have introduced numerous cultivation and composting techniques along with crops requiring less space and water, the restrictions in time and space were mentioned as the main reasons against not practicing composting or gardening. The present findings also agree with a previous study conducted in Kandy and the limited interest raises a question on whether the community is actually moving towards environmentally friendly lifestyles and vector management or not [14]. Collection of waste by the Municipality or Urban Council remained as the major waste disposal method followed by open burning and disposal into a garbage pit. As emphasized by Gubler & Clark [52], properly planned urbanization and waste disposal services are key infrastructural features that minimize the incidence of dengue outbreaks. Unfortunately, the irregular nature of the collection date, rejection of certain items, use of no alarming sound to inform the residents that they are visiting the area and not reaching certain roads or houses, were found as major weaknesses of the waste collection service, (especially among dengue impacted households), that may have clearly contributed to the elevated risk of dengue within the study areas [33, 52, 53]. Therefore, implementation of a proper functioning system for waste collection is recommended, which is more user-friendly and caters for the requirements of the community to ensure proper waste management in the study areas, thereby assisting the management of dengue vector breeding.

Regarding practices, the use of mosquito coils and creation of smoke were more common preventive measures practiced against mosquito–biting, followed by the use of nets and closing/covering of windows. Another study conducted in Jamaica [47], has reported that approximately 80% of study participants were not using any effective preventive methods such as mosquito screening and bednets, due to higher costs of implementation. Conversely, Sri Lankan communities were rationally utilizing available resources to minimize and prevent bites of vector mosquitoes at their households, by incorporating some of the traditional methods. Use of coconut husks, cashew shells and dry leaves of plants to create smoke to avoid mosquito bites were highly practiced in many households (especially in Kandy), due to their low economic costs and high efficacy with limited side-effects. Other studies conducted in Pakistan [54] and Mexico [55] have also found the same tendency of using mosquito coils and screens as methods of mosquito bite prevention and have reported their successful contributions in minimizing the severity of dengue outbreaks.

Covering water containers/water storage tanks and eliminating potential breeding sites of vector mosquitoes were recognized as the most common practices of the community. A number of studies have documented such preventive practices to be practiced by different communities all over the world [13, 14, 47, 48, 54]. However, proper disposal of solid waste, organizing “Shramadana” (clean-up programmes) to clean the surrounding and clearing bushes and other vegetation were practiced more by the healthy populations. According to studies conducted in Pakistan [54] and Thailand [50], implementation of clean-up programmes often is highly effective in controlling dengue transmission, especially if organized either prior to or at the beginning of the rainy season. Such practices often ensure the unity and social responsibility of the community residing at the ground level, which may be further improved and converted into community-based vector management strategies with the guidance and support from other government and private sector stakeholders. Several studies have highlighted the importance of driving the common public towards elimination of vector breeding sites at the household level *via* raising awareness as a successful solution for managing the transmission of dengue [13, 47, 50]. Therefore, regardless of the moderate to high knowledge levels of the community on various aspects of dengue, the preventive practices and attitudes requires further improvement to ensure a dengue-free environment, as highlighted by several similar studies [47, 56].

Based on the above, it is ostensible that the people residing in dengue-free households have a relatively higher degree of knowledge and more helpful attitudes toward dengue along with more preventive practices to ensure minimum levels of dengue vector breeding and human-vector contact. Comparatively, the patient groups of both districts had limitations in their awareness, social status, attitudes on dengue and practices against dengue. In particular, the knowledge on dengue symptoms, patient care, vector biology and behavior should be further improved. Therefore, the relevant VCE should design their awareness programmes to cater to the above requirements of the community and effective knowledge transmission methods should be followed to address the limitations in knowledge on different aspects of dengue. Improving knowledge on symptoms and treatment methods of dengue within the community will drastically reduce their reliance on traditional remedies and self-medication, while driving them towards immediate hospitalization. This would be immensely helpful for the VCE and other health staff in Sri Lanka, not only for the management of patients but also to minimize patient-based transmission of the virus to other vectors and thereby to humans. Regarding attitudes, the public should be made aware that the responsibility of managing dengue epidemics at the local level should be equally borne by the government based VCE, private stake holders and also by the general public to ensure efficacy of any intervention actions.

Furthermore, VCE and other government staff should work alongside the community to win the trust of the general public regarding the adequacy of their services provided in managing dengue, which was found to be poor. Routine inspections of the households for dengue vector breeding sites, organizing cleaning and awareness programmes (particularly before onset of rainy season) and facilitating local vector management activities organized by the community or other organizations are key steps to be followed by the VCE, to motivate the public towards community-based management of dengue [57]. In addition, local administrative bodies such as Municipal Councils and “Pradeshiya Sabha” (village councils), should provide key infrastructure facilities such as properly planned urbanization and waste disposal services, taking necessary steps to avoid weaknesses such as the irregular nature of the collection date, rejection of certain items, use of no alarming sound to inform the residents that they are visiting the area and not reaching certain roads or houses during waste collection. This would also motivate the public in practicing source separation of solid waste and proper disposal of solid waste, minimizing the potential of vector breeding. In addition to the chemical-based controlling of vector breeding, the VCE should encourage the public to move towards traditional methods of mosquito-human contact reduction such as use of coconut husks, cashew shells and dry leaves of plants to create smoke at dawn and just before dusk.

The findings of the study highlight the high susceptibility of males belonging to the 15–35 age category, who spend much of the day outside. The VCE should therefore identify that the risk of human-vector contact may arise from public environments such as schools, tuition classes, working places and bus stands etc., which are poorly managed in terms of vector breeding reduction. As solutions, the VCE could establish strong connections with the other government administrative entities (such as Municipal Councils and regional educational offices), the private sector and also with religious leaders of the locality to coordinate vector controlling activities and ensure vector-free environments in public places.

The recent outbreaks of dengue that have occurred since the beginning of 2017, simulated different parties such as Government VCE, NGOs and other private stakeholders to focus more on dengue while communicating the risk to the common public. This could be the reason for the elevated levels of knowledge, attitudes and preventive practices of the common public on different aspects of dengue. However, a number of studies including the WHO and CDC have recommended community-based vector management as the potential solution for the management of dengue, rather than relying upon conventional methods of chemical based control [11–13, 33, 47, 57]. Therefore, the VCE of Sri Lanka should focus more on bridging the gap in knowledge and attitudes on dengue among the general public, motivating them to work with other stakeholders to ensure personal and community-wise protection from dengue. As such, it is essential to design community educational campaigns to educate residents on different aspects related to dengue, while emphasizing the responsibility of the community in vector management, to ensure community-based controlling of dengue within the country [13, 14, 33, 57]

## Conclusions

In conclusion, the findings of the present study highlight the importance or further improvement in community education and motivation. More importantly, proper communication and coordination between different entities working on dengue and the local communities to recognize key constrains/practical difficulties in dengue disease management is required. In addition, the key infrastructural risk factors such as properly planned urbanization and proper waste collection, etc. should be further improved. In spite of the limitations of the present study, it could be recommended that the government based VCE should focus more on the actual conditions represented by the public on knowledge, attitudes and personal protective practices as presented by the research-based findings. Hence, the present study warrants that health authorities should design flexible and community-friendly intervention programmes to ensure efficacy and sustainability of such control programmes through community-based integrated vector management strategies.

## Abbreviations

CDC: Centers for Disease Control and Prevention
IVM: Integrated Vector Management
MOH: Medical Officer of Health
PHI: Public Health Inspector
VCE: vector controlling entities
SWM: Solid Waste Management
WHO: World Health Organization
